# Untargeted LC-HRMS Based-Plasma Metabolomics Reveals 3-O-Methyldopa as a New Biomarker of Poor Prognosis in High-Risk Neuroblastoma

**DOI:** 10.3389/fonc.2022.845936

**Published:** 2022-06-10

**Authors:** Sebastiano Barco, Chiara Lavarello, Davide Cangelosi, Martina Morini, Alessandra Eva, Luca Oneto, Paolo Uva, Gino Tripodi, Alberto Garaventa, Massimo Conte, Andrea Petretto, Giuliana Cangemi

**Affiliations:** ^1^ Chromatography and Mass Spectrometry Section, Central Laboratory of Analysis, IRCCS Istituto Giannina Gaslini, Genoa, Italy; ^2^ Core Facilities Clinical Proteomics and Metabolomics, IRCCS Istituto Giannina Gaslini, Genoa, Italy; ^3^ Clinical Bioinformatics Unit, IRCCS Istituto Giannina Gaslini, Genoa, Italy; ^4^ Laboratory of Molecular Biology, IRCCS Istituto Giannina Gaslini, Genoa, Italy; ^5^ DIBRIS, University of Genoa, Genoa, Italy; ^6^ Department of Pediatric Oncology and Hematology, IRCCS Istituto Giannina Gaslini, Genoa, Italy

**Keywords:** neuroblastoma, metabolomics, biomarker, high resolution mass spectrometry, catecholamines, 3-O-methyldopa

## Abstract

Neuroblastoma (NB) is the most common extracranial malignant tumor in children. Although the survival rate of NB has improved over the years, the outcome of NB still remains poor for over 30% of cases. A more accurate risk stratification remains a key point in the study of NB and the availability of novel prognostic biomarkers of “high-risk” at diagnosis could help improving patient stratification and predicting outcome.

In this paper we show a biomarker discovery approach applied to the plasma of 172 NB patients. Plasma samples from a first cohort of NB patients and age-matched healthy controls were used for untargeted metabolomics analysis based on high-resolution mass spectrometry (HRMS). Differential expression analysis highlighted a number of metabolites annotated with a high degree of identification. Among them, 3-O-methyldopa (3-O-MD) was validated in a second cohort of NB patients using a targeted metabolite profiling approach and its prognostic potential was also analyzed by survival analysis on patients with 3 years follow-up. High expression of 3-O-MD was associated with worse prognosis in the subset of patients with stage M tumor (log-rank p < 0.05) and, among them, it was confirmed as a prognostic factor able to stratify high-risk patients older than 18 months. 3-O-MD might be thus considered as a novel prognostic biomarker of NB eligible to be included at diagnosis among catecholamine metabolite panels in prospective clinical studies. Further studies are warranted to exploit other potential biomarkers highlighted using our approach.

## Introduction

Neuroblastoma (NB) is the most common extracranial malignant tumor in children. Although the survival rate of NB has improved over the years, the outcome of NB still remains poor in about 30% of cases (1). Patient prognosis is currently based on a combination of clinical, histopathological and biological features such as age at diagnosis, stage of the disease, MYCN amplification, loss of heterozygosity for chromosome 1p and 11q (LOH1p and LOH11q), tumor ploidy established at diagnosis. On these bases, patients are classified in different risk groups and addressed to different treatment protocols (2). An age of 18 months is considered as the cutoff age distinction for most of the risk stratification, as children older than 18 months at diagnosis typically have worse outcomes. Patients with oncogene MYCN amplification are classified as high-risk. In addition, any patient with metastatic disease and age 18 months or older is considered at high-risk irrespective of MYCN amplification. A more accurate risk stratification remains a key point in the study of NB as patients receiving the same treatment can still have a markedly different clinical course. In particular, the availability of novel prognostic biomarkers of high-risk NB at diagnosis could help in improving patient stratification, accurately predicting outcome, relapse or response to treatments and also reducing unnecessary therapies and related toxicities. Many efforts have been made to identify novel prognostic biomarkers using different Omics approaches based on gene expression analysis (3). An alternative emerging tool for biomarker discovery and personalized medicine is represented by metabolomics. Metabolomics has the great potential to elucidate the ultimate products of the genomic processes that lead to altered metabolism, that is one of the defining features of cancer (4). Over the last decade, the combination of untargeted and targeted metabolomics approaches has greatly facilitated the discovery of many cancer biomarkers with prognostic potential, such as prostate, breast and colorectal cancer (5–7). A powerful instrument increasingly used to produce metabolomics data for the identification and quantification of compounds is high resolution mass spectrometry (HRMS) (8, 9). By providing mass measurements with high resolution and accuracy, HRMS allows the analysis of complex matrices and the potential detection of hundreds metabolites (10). A key point of metabolomics is the development of robust workflows for compound identification, data analysis and biological interpretation (11). Previous metabolomic analyses conducted on murine xenograft models with nuclear magnetic resonance (NMR) (12) or plasma of NB patients by LC-MS (13) support the potential of metabolomic profiling for improving NB risk-group stratification and outcome prediction. Nevertheless, these studies were not able to provide candidate biomarkers with an independent validation in a second cohort of patients.

In this paper, an HRMS-based approach was applied to understand the dynamic metabolic modifications associated with NB and to measure the abundance of metabolites in patients’ plasma with the goal of discovering prognostic biomarkers that could help improving patient stratification. Starting from an untargeted approach the differences in the metabolomic profiles between groups of patients were investigated by differential expression analysis. Among the significant metabolites of the L-DOPA degradation pathway 3-O-methyldopa (3-O-MD), also known as 3-Methoxytyrosine (PubChem CID: 1670, CAS: 7636-26-2), was selected and validated in a second cohort of patients using a targeted approach based on liquid chromatography coupled to tandem mass spectrometry (LC-MS/MS) and its prognostic value was dissected.

## Materials and Methods

### Patients and Samples

The study included a total of 172 patients with histologically confirmed NB. A written consent allowing the collection of samples and the use of clinical and nongenetic data for clinicalresearch was signed by the patient’s guardians. The study was approved by the Regional Ethical Committee (ANTECER_Neuroblastoma 16/09/2019).

All NB patients were diagnosed between 2011 and 2018 in 29 institutions belonging to the Associazione Italiana di Emato-Oncologia Pediatrica. NB patients were assigned to the different risk groups according to the International Neuroblastoma Risk Group (INRG) pre-treatment risk schema and addressed to the different treatment protocols (2). Fasting plasma samples were obtained from peripheral venous blood collected in 3 mL EDTA K3-containing tubes, centrifuged at 4000 g for 5 min at 4°C and stored at -80°C until analyzed. Leftover plasma samples of outpatients after routine clinical analyses were used as healthy controls.

Samples were divided in two sets: the first set (n=99) was used for biomarker discovery purposes while the second set (n=122) was used for biomarker validation. The first set of samples was obtained from 27 control subjects (CTR) and 50 NB patients among which 28 patients with localized NB (INRG L1, L2) or MS and 22 with metastatic NB (INRG M) which have been sampled at two timepoints: at diagnosis and after induction chemotherapy. The second set consisted of 122 samples from 69 patients with localized NB and 53 with metastatic NB (INRG M). **Table 1** summarizes the clinical demographics of the two sets.

**Table 1:** Clinical characteristics of the patients and the control subjects included in the study.

	First cohort (n=77)				Second cohort (n=122)			
	Patients		Controls		Patients		Controls	
	n	%	n	%	n	%	n	%
**Gender**								
M	21	42.0	12	54.5	56	51.3	0	0
F	29	58.0	10	45.5	66	48.7	0	0
**Age at diagnosis**								
<18 months	21	42.0			53	43.4	0	0
>=18 months	29	58.0			69	56.5	0	0
**INSS stage**								
L1	25	50.0	0	0	11	9.0	0	0
L2	2	4.0	0	0	51	41.8	0	0
M	22	44.0	0	0	53	43.4	0	0
MS	1	2.0	0	0	7	5.7	0	0
**MYCN status**								
Amplified	11	22.0	0	0	28	22.9	0	0
Not amplified	30	60.0	0	0	87	71.3	0	0
na	9	18.0	0	0	7	5.7	0	0
**Event overall**								
yes	2	4.0	0	0	20	16.3	0	0
no	48	96.0	0	0	100	81.9	0	0
na	0	0.0	0	0	2	1.6	0	0
**Overall survival**								
mean (std dev)	455 (395)		0	0	1111 (369)		0	0

In the first set, sex (male vs female) and age group (<18 months vs >18 months) were tested for confounding between CTR subjects and NB patients using Fischer’s exact test; P value less than 0.05 was considered significant (**Supplementary Table 1**).

### Sample Preparation for Untargeted Metabolomic Analyses

Plasma samples used for untargeted metabolomic analyses were prepared as follows: a 50 µL aliquot of plasma was extracted adding 150 µL of cold (-20°C) methanol containing an internal standard (IS) mixture (**Supplementary Materials, 1.1** Chemicals and consumable) and centrifuged for 10 minutes at 14,000 x g at 4°C. The supernatant was then collected and stored at -80°C until analyzed. A 50 µL aliquot of supernatant was then added to an equal volume of IS kit (MSK-QC-KIT) purchased by Cambridge Isotope Laboratories, Inc (Tewksbury, MA, USA) (**Supplementary Materials, 1.1** Chemicals and consumable), vortex mixed and 5 µl were injected in the UHPLC system. Quality control (QC) samples were prepared by pooling together supernatants obtained from all the samples; external quality control (EQC) samples were prepared by pooling together plasma obtained from healthy adult volunteers extracted as described above.

### Untargeted HRMS Metabolomic Analyses

LC-HRMS analysis was carried out using a Vanquish Horizon UHPLC system coupled to a Q-Exactive Plus Hybrid Quadrupole-Orbitrap Mass Spectrometer (Thermo Fisher Scientific, Milan, Italy) as previously described (11). The liquid chromatographic separation was carried out using two different chromatographic conditions: reversed phase and hydrophilic interaction liquid chromatography (HILIC) (**Supplementary Materials, 1.2** Untargeted metabolomic analysis). Ionization was obtained using a heated electrospray source probe both in positive and negative mode with spray voltages at 3.9 kV and 3.7 kV, respectively. The capillary temperature was set at 300°C. Nitrogen sheath and auxiliary gas were set at a flow rate of 35 and 5 arbitrary units respectively. Extracted samples were splitted into four injection batches, and processed combining the four different conditions mentioned above (two different chromatographic conditions and two polarities). For each batch, analyses were performed using two different acquisition modes with an m/z range 70–1200. The first acquisition mode, used for compound profiling purposes, was a full MS scan mode with the following parameters: resolution of 70000, auto gain control target < 1 × 10^5^ and maximum injection time 100 ms. The other acquisition mode, used for identification of unknown compounds, was a full MS scan acquisition followed by data-dependent fragmentation (MS2) scan (DDA) with a resolution of 17500, an auto gain control target of 1 × 10^5^, a maximum injection time of 65 ms, a loop counts of top 5 peaks and an isolation window of m/z 1.2. All MS2 spectra of the compounds were acquired at 3 collision energies: 20, 40 and 80 eV. The MS1 mass range was divided into 10 mass ranges with a width of 50 m/z each on which the data dependent analysis was carried out. DDA was performed with a priority fragmentation for the m/z of our Accurate Mass Retention Time (AMRT) library (11).

The run order used to perform the metabolomic analyses started with 5 procedural blank samples composed by the solvents used during all steps of sample preparation, for contamination monitoring and system conditioning purposes; 18 QCs analyzed in DDA mode, for identification purposes, followed by the runs of the 99 samples that were randomized in the analytical sequence to avoid bias due to instrument drift; finally, 16 QCs and 9 EQCs added every 6 and 12 runs respectively. The experimental design and the analytical workflow are summarized in the Supplemental figures (**Supplementary Figure 1**).

### Data Processing, Metabolite Identification and Pathway Analysis

MS1 full-scan .RAW files have been converted into .ABF files using ABF converter (14) and processed with MS-DIAL ver.4.24 software (15) for deconvolution, peak picking, alignment and compound identification. Compounds, initially, were identified by matching retention times, accurate precursor masses, and MS/MS spectra with a previously published AMRT database (11) to provide a level 1 identification of metabolites (16). For MS2 spectra and precursor mass matching, the freely available library MassBank of North America (17) and mzCloud (18) were also used. The four tables obtained by MS-DIAL, generated combining the two different chromatographic methods and the two different MS polarities were exported as a .txt file.

The dataset, composed of four different sample sets and QCs was imported and statistically analyzed in Perseus (19). The QC variation coefficient was calculated and then used to exclude features variations > 0.4. Subsequently, the expression data was log2 transformed and the QC group removed. Each feature was, therefore, filtered by row with a valid value of at least 70% for each group. The missing data were replaced by the QRILC method, which performs the imputation of left-censored missing data, using random draws from a truncated distribution with parameters estimated by quantile regression. Quantile normalization was instead used to normalize the intensity values which were further filtered with the MS/MS assigned category, considered necessary to continue the analysis. Indeed, for the annotations of in silico compounds, starting from ionic features with associated MS2 events, MS-FINDER ver.3.26 was used (20). The MS-FINDER annotation matrix was merged with the quantitative matrix by employing the matching row by name Perseus option and the Alignment ID was used as the unique classifier. A MS-FINDER Structure Rank score > 5 and a mzCloud identification score > 80 were used to select features that were worthy of statistical investigation.

A differential analysis was then performed using the Limma Test package, the parameters are the same for all chromatographs and use the Voom option for data editing with a span of 0.5, normalization is quantile, with an acceptability threshold for up-regulation and down-regulation of 2 with respect to a Log2 Fold Change and an adjusted p-value (FDR) of less than 0.05.

Compounds with a Human Metabolome Database (HMDB) accession were matched with the HMDB database (“All Metabolites’ ‘ file dated 2021-10-23) (21) to add categorical information to the expression matrix like associated proteins. Statistically significant metabolites (and associated proteins) are used in enrichment analyses such as in ClueGO (22), a Cytoscape App (23), and MetaboAnalyst (24) to visualize their associated pathways.

### Targeted Analysis of 3-O-Methyldopa by LC-MS/MS

Targeted quantitative analysis of 3-O-MD was performed on the second set of patients by LC-MS/MS on a TSQ Quantiva mass spectrometer coupled to an Ultimate 3000 UHPLC (Thermo Fisher, Scientific, Milan, Italy). Briefly, 50 µL of plasma were subjected to protein precipitation with 150 µL cold (-20 ∘C) methanol containing 3-o-methyldopa-d3 as internal standard. A 5 µl aliquot of the supernatant was then injected in the LC-MS/MS system using a gradient separation chromatography. Method performance was validated following EMA guidelines for bioanalytical method validation (25) (**Supplementary Materials, 1.3** Targeted analysis of 3-O-methyldopa by LC-MS/MS and 1.4 Mass spectrometry methods validation and quality assurance).

### Statistical Analysis

Overall survival (OS) curves were plotted by the Kaplan-Meier method and compared with the log-rank test using GraphPad Prism ver. 8 (26). For improving reliability, survival analysis was performed on alive patients with at least 3 years of follow-up. Patients lost at the follow-up or with missing survival information were excluded from the analysis. Cutoff values distinguishing between low or high 3-O-MD concentrations levels were identified by Elbow method (27), which is an empirical method that, given a numerical variable, selects one or more cut-off values whose slope change is evident. Significance of the expression differences of 3-O-MD across tumor stages was assessed by one-way ANOVA test. Post-hoc analysis was performed by Tukey’s multiple comparisons test.

## Results

### Untargeted Metabolomic Profiling of NB

**Figure 1:**
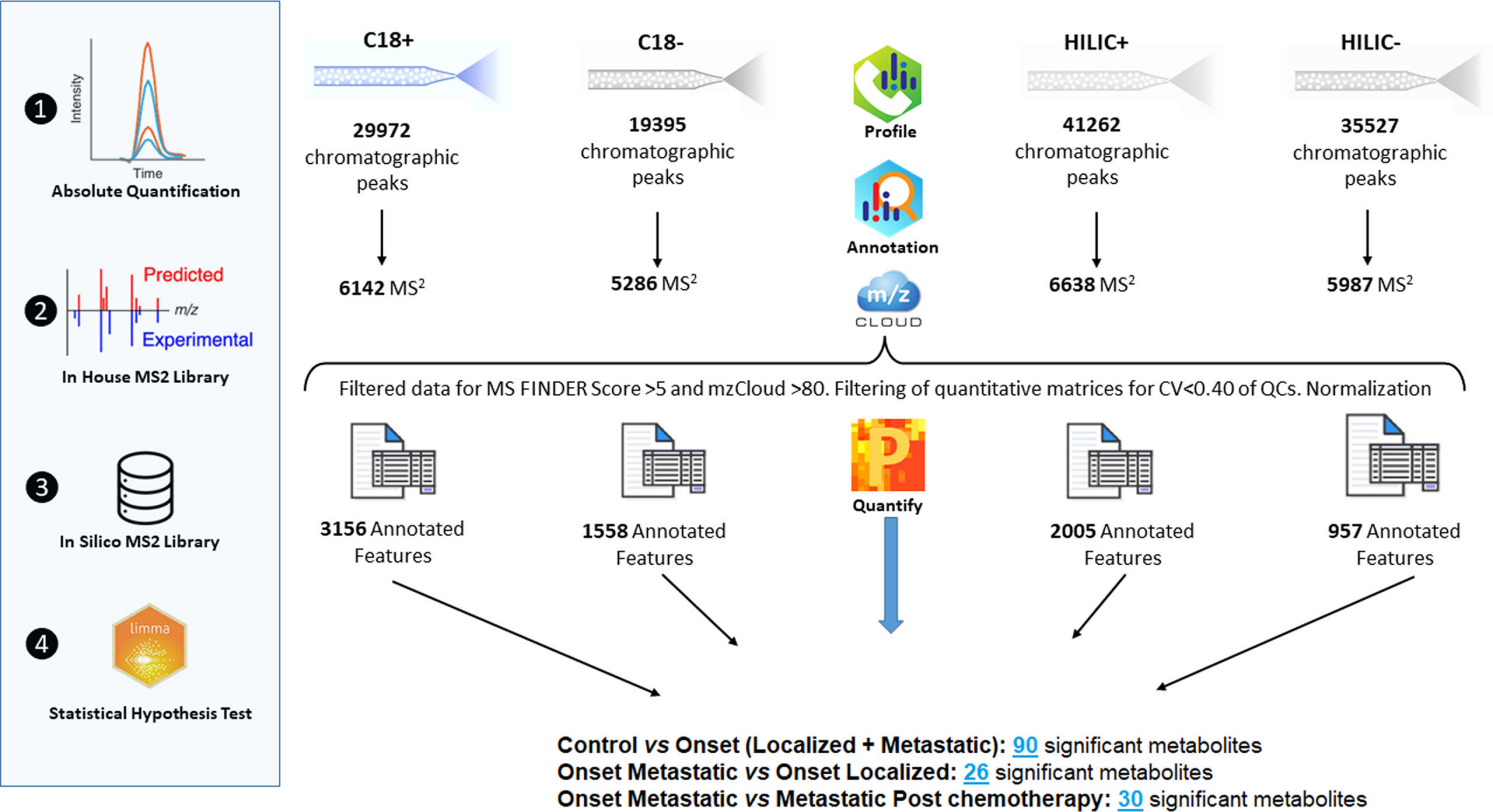
Overview of raw data manipulation, reduction of the number of features queried by statistical analysis.

Raw data were deposited in MetaboLights Data Repository (28) and assigned the identification code MTBLS4294. A total of 99 metabolic profiles were obtained from plasma samples of the first cohort from untargeted metabolomic analyses and metabolite identification as described in previous paragraphs. Filtering of data resulted in a significant reduction of the number of features to be queried by statistical analysis (**Figure 1**). Features were then selected for CV, retention times and MS2 annotation. Quantitative differences in metabolites expression were detected across different groups of patients and controls by using the Limma test. The statistically significant metabolites were annotated with related proteins and metabolic pathways in order to understand their involvement in biological processes (**Supplementary Figure 2**). The expression of the metabolism of Tyrosine, dopamine and catecholamines should be highlighted. The main reactions of the metabolic process take place through the activity of, among others, the enzymes Catechol-O-Methyltransferase, Aldehyde Dehydrogenase and Monoamine Oxidase (**Supplementary Figure 3** and **Supplementary File 1**). Another path that is important to mention is the metabolism of Arginine and Proline. Recent studies on cancer metabolism have highlighted the role shown by proline metabolism, in particular its critical role in cancer reprogramming and its clinical relevance (29).

In order to estimate possible confounding effects between CTR subjects and NB patients, we studied the association between sex (M vs F) or age group (<18months vs >18 months) and subject group (CTR vs NB). Fischer’s exact test p value did not show a significant association between sex or age group and the subject group (p>0.05), thereby excluding the possibility that sex or age might be confounding factors in our study.

**Figure 2:**
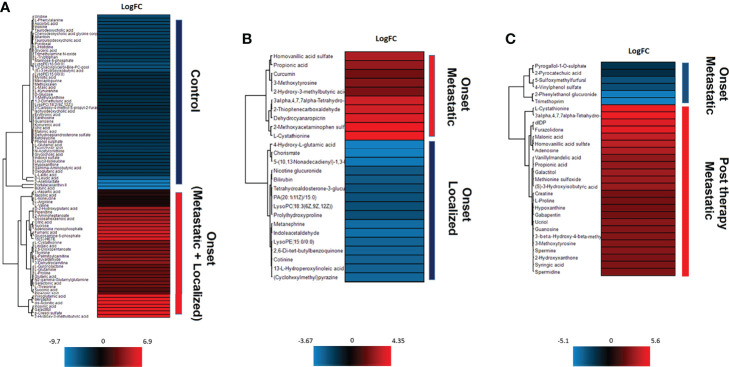
Unsupervised hierarchical-clustered heatmap of metabolites identified by t-test in the comparison between: **(A)** NB patients at the onset and healthy controls. **(B)** metastatic and localized NB patients at the onset. **(C)** metastatic NB patients at the onset and after chemotherapy.

As shown in **Figure 2A**, the differential expression analysis between samples from NB patients at the onset and controls revealed the presence of 90 significantly modulated metabolites (**Supplementary Table 2**) belonging to different metabolic pathways, among which L-cystathionine and 2-Hydroxy-3-methylbutyric acid were evident. The comparison between localized (INRG stage L1 and L2) and metastatic (INRG stage M) patients at diagnosis highlighted 26 significantly modulated metabolites (**Supplementary Table 3** and **Figure 2B**). In particular, Homovanillic acid sulfate and 3-O-MD, metabolites involved in the L-DOPA metabolism, were found to be overexpressed in metastatic patients. On the contrary, Metanephrine resulted significantly down expressed in metastatic patients. Noticeably, in metastatic patients, L-cystathionine and 2-Hydroxy-3-methylbutyric acid were overexpressed. As shown in **Figure 2C**, the differential expression analysis using samples from metastatic patients at diagnosis and after induction chemotherapy highlighted the significant modulation of 30 metabolites (**Supplementary Table 4**) and pointed out the expression alteration of three metabolites of L-DOPA metabolism pathway: 3-O-MD, Homovanillic acid sulphate and Vanillylmandelic Acid, which resulted upregulated in metastatic condition. Moreover, L-Cystathionine and 3-Hydroxyisobutyric acid were also overexpressed at diagnosis, as well as, a number of metabolites of the methionine metabolism (adenosine, L-cystathionine, methionine sulfoxide and spermidine) and polyamines (Spermine and spermidine).

Since 3-O-methyldopa was found significantly modulated in several comparisons and is a metabolite of the catecholamine pathway mentioned in literature (30), we decided to focus on it in subsequent analyzes.

### Validation of 3-O-Methyldopa as Potential Clinically Relevant Biomarker for NB

**Figure 3:**
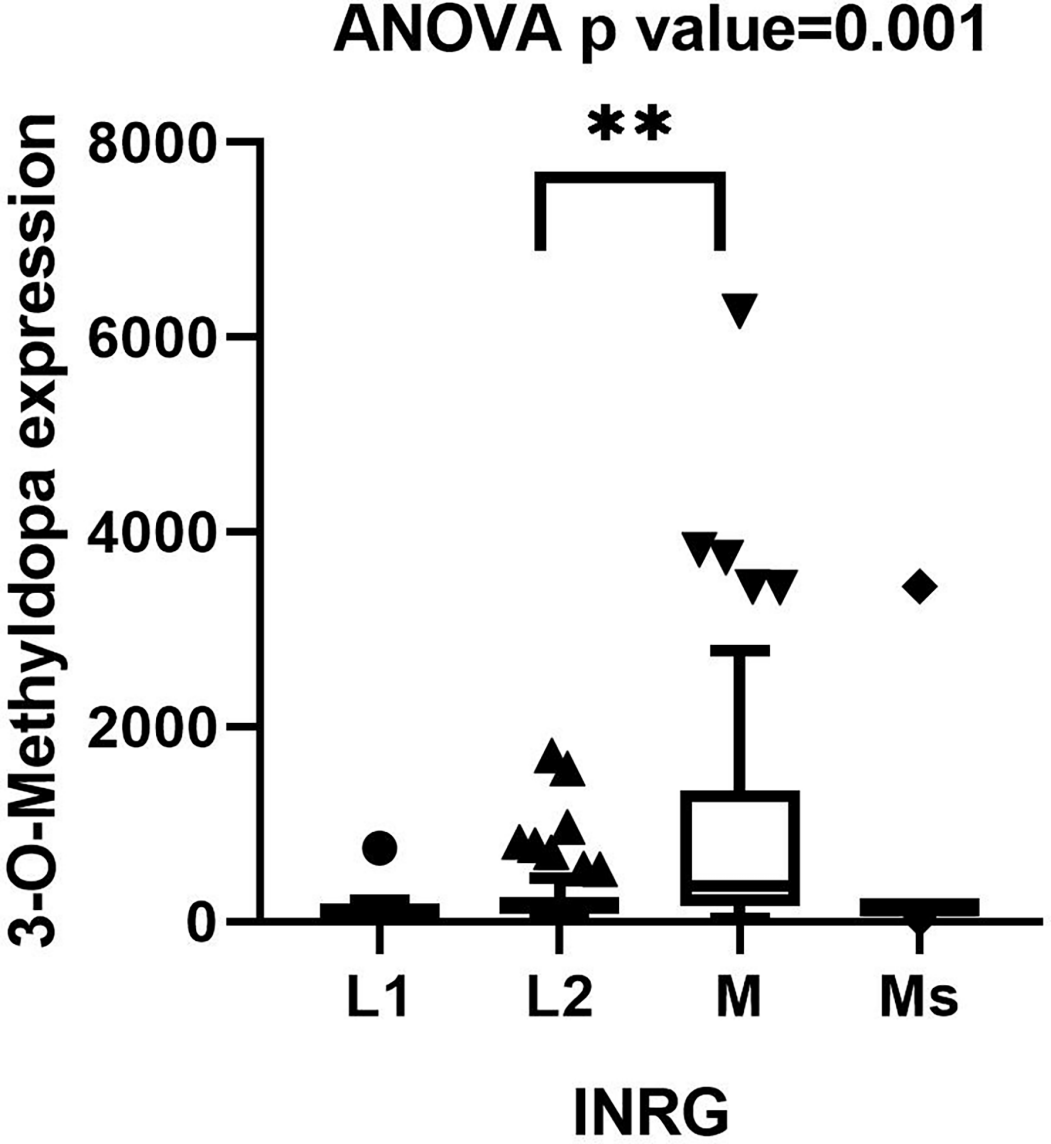
Boxplot displaying the distribution of 3-O-Methyldopa expression in 122 NB patients grouped by INRG stage. Boxplot was visualized using Tukey’s method. Significance of the expression differences of 3-O-Methyldopa across INRG stages was assessed by one-way ANOVA test. P value is reported on top of the panel. Post-hoc analysis comparing 3-O-Methyldopa expression between every possible pair of INSS stages was performed by Tukey’s multiple comparisons test. Significant pairs were indicated by brackets and asterisks. ** indicates p value lower than 0.005.

SupplementalThe concentrations of 3-O-MD by LC-MS/MS ranged between 23.6 and 6272.8 ng/ml. In order to validate the association between 3-O-MD expression and metastatic NB that was observed in our untargeted analysis, the distribution of 3-O-MD expression was reported across the subsets of patients defined by the INRG stage (**Figure 3**). One-way ANOVA was used to estimate the significance of the 3-O-MD expression differences across stages. Post-hoc analysis using Tukey’s correction method was used to identify groups of patients with significantly different 3-O-MD expression. ANOVA test showed significant 3-O-MD expression differences across stages (ANOVA p value<0.05). Post-hoc analysis showed a statistically significant difference between stage M and stage L2 tumor subsets (p<0.05), thereby confirming our findings on the first cohort of NB patients. Visual inspection of the boxplot showed a higher 3-O-MD expression in the subset of stage M tumors with respect to other tumor stages (**Figure 3**).

Since NB patients with stage M tumors are characterized by a disseminated disease and poor prognosis (2), we hypothesized a potential prognostic value of 3-O-MD at the onset for NB.

**Figure 4:**
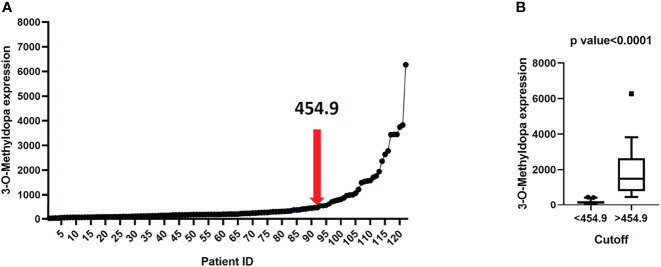
3-O-Methyldopa expression cutoff visualization. **(A)** Scatter plot reporting 3-O-Methyldopa expression in 122 NB patients. Cutoff, selected by the Elbow method, is reported within the plot. The relative rank position of the cutoff is shown by a red arrow. **(B)** Boxplot displaying the low or high 3-O-Methyldopa expression in 122 NB patients according to the cutoff value. Boxplot was visualized using Tukey’s method. Significance of the expression difference between low and high 3-O-Methyldopa expression was carried out by unpaired student’s t test. P value is reported on top of the panel.

To estimate whether NB patients might be divided into groups on the basis of 3-O-MD expression, we split patients into two groups using the Elbow method (27), which was able to return the cut-off point from an ordered numerical variable. The application of the Elbow method to 3-O-MD expression identified 454.9 as a candidate cut-off value (**Figure 4A**). This cut-off value divided the cohort into two groups of 91 and 31 patients with high or low expressions of 3-O-MD, respectively. Unpaired t test assessed the significance of 3-O-MD expression difference between the newly defined groups of patients. The analysis highlighted a statistically significant difference of 3-O-MD expression between the two groups of patients (p<0.0001, **Figure 4B**) and confirmed that NB patients may be divided into groups based on 3-O-MD expression.

**Figure 5:**
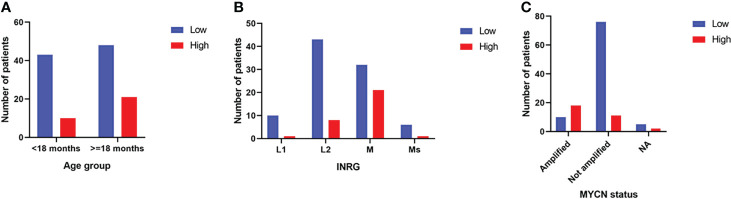
Bar plots of the distribution of 3-O-Methyldopa expression in the second cohort. The bar plots show the number of patients with low (blue) or high (red) 3-O-Methyldopa expression in the subsets of patients defined by: **(A)** age at diagnosis, **(B)** INRG stage and **(C)** MYCN status. Age at diagnosis was split into two groups, one >=18 months and the other <18 months to simplify the analysis. NA stands for not accessible value. Low and high 3-O-Methyldopa expression is displayed in the legends.

The distribution of the two populations of patients with low or high 3-O-MD expression in the subsets defined by age at diagnosis (<18 months vs. > 18 months), INRG stage, and MYCN status (amplified vs. not amplified) was then evaluated. The number of patients with high 3-O-MD expression was greater than zero in all subsets except for patients with stage L1 tumor, but it was higher in the subsets of patients older than 18 months, INRG stage M, and amplified MYCN tumors (**Figure 5**). These results indicate that high 3-O-MD expression is associated with unfavorable clinical characteristics. Furthermore, a non-trivial number of patients older than 18 months, INRG stage M, and amplified MYCN tumors had a low 3-O-MD expression suggesting that 3-O-MD may additionally stratify these subsets of patients. In order to test this hypothesis, we assessed the stratification of selected subsets of patients defined by known prognostic markers.

**Figure 6:**
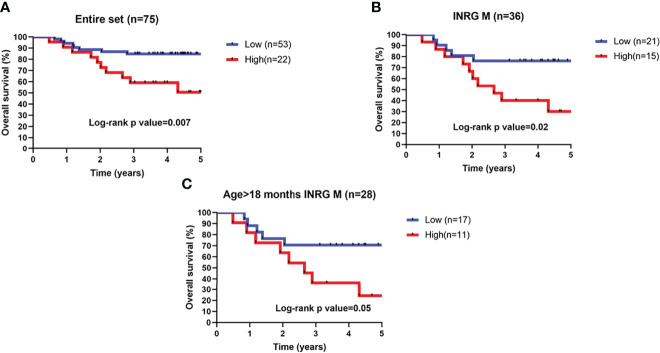
Kaplan–Meier estimates and significance of NB patient OS by 3-O-Methyldopa expression. Kaplan-Meier curves show OS of NB patients with high (red) or low (blue) 3-O-Methyldopa expression in a time interval of 5 years. OS is displayed in years. To enhance reliability, data include alive patients with at least 3-years of follow-up. Low or high 3-O-Methyldopa expression was determined according to a cutoff of 454.9 determined with the Elbow method for the second dataset. Plots are relative to **(A)** All patients, **(B)**, stage M tumors and **(C)** high-risk patients older than 18 months with stage M tumor. Plots are entitled with the characteristics of the patients in the sub-population. Survival curves were compared by log-rank test. The number of patients with low or high 3-O-Methyldopa expression is reported within brackets in the legend.

In order to improve analysis reliability, survival analysis was performed on alive patients with at least 3 years of follow-up excluding from the analysis patients lost at the follow-up or with missing survival information. Data from remaining 75 patients were then used for subsequent analyses. High or low 3-O-MD expression was able to stratify the entire cohort of 75 patients into groups with significantly different overall survival (log-rank p < 0.05, **Figure 6A**). High expression of 3-O-MD metabolite was associated with worse prognosis indicating that 3-O-MD can be considered as a new unfavorable prognostic factor for NB.

Next, the prognostic value of 3-O-MD was assessed in additional clinically relevant subgroups of patients defined by established prognostic markers. Specifically, 3-O-MD significantly stratified patients with stage M tumors, in which, high expression of 3-O-MD metabolite was associated with worse prognosis (log-rank p < 0.05, **Figure 6B**).

We then assessed the clinical utility of 3-O-MD in the subset of high-risk patients older than 18 months with stage M tumor that are difficult to be stratified with actual risk factors (31) and we found that 3-O-MD was able to significantly stratify them (log-rank p ≤ 0.05, **Figure 6C**).

Our findings highlighted the ability of 3-O-MD to stratify clinically relevant subsets of patients, thus supporting the clinical utility of 3-O-MD metabolite for NB.

## Discussion

Metabolomics is a powerful tool for the identification and quantification of small molecule metabolic products of a biological system. It has been successfully exploited in different pathological contexts such as breast cancer (6), colorectal cancer (7), cardiovascular diseases (32, 33), down syndrome (34), celiac disease (35) among others. The analytical techniques most commonly used for metabolomics are: NMR and LC-MS.

Metabolomics is revealing its potential for biomarker discovery in complex diseases and may provide new information to understand disease pathology (36).

In this paper, we show the application of a HRMS metabolomic approach to NB. Other authors have previously described metabolomics studies on NB using NMR (12) or LC-MS (13). Both papers confirmed the potential of metabolomic approaches applied to NB. Beaudry et al. (12) were able to identify distinctive metabolic changes of NB using mouse models, but their results have been carried out on a very small numbers of patients’ samples therefore not comparable with our study.

Morover, it is interesting to note that Quintás et al. (13) were able to identify distinct plasma profiles in high-risk and low-risk patients at diagnosis and the ability of metabolomics to potentially predict patients who are more likely to progress during treatment. As discussed below, our approach was able to identify some metabolites that were thus confirming the reproducibility of their study. Our work, however, went a step further by validating on a second independent cohort of patients one of the most statistically significant and promising metabolites.

In this paper, we demonstrated that starting from a very small (50 µL) amount of plasma HRMS-based metabolomics is able to distinguish between healthy subjects and patients with NB and to improve the actual risk stratification of NB patients.

Using a completely unbiased approach and a randomized order of analysis of samples, we were able to confirm the presence of key metabolites of the DOPA catabolic pathway, which has been known to be dysregulated in NB since the late 70’s (37). In fact, it has been demonstrated that O-methylated catecholamine metabolites in urine possesses high diagnostic sensitivity rates in NB (81.9-85%) (38). Moreover, catecholamine excretion patterns have been correlated with different NB features and outcome, underlying potential distinguishing metabolite that may help in the assessment of risk-stratification (39, 40). In plasma, the few data available on catecholamine metabolites in NB (41–43) suggest comparable conclusions found in urines, with a higher presence of early metabolites of the DOPA catabolic pathway associated with a more aggressive behavior of the tumor.

In the present study, differential expression analysis from plasma samples of patients and healthy controls identified metabolites, including 3-O-MD, which was found significantly more expressed in samples from metastatic NB patients than those with localized tumors at the onset of the disease.

3-O-MD, is a direct metabolite of L-DOPA and is produced by the enzyme catechol-O-methyltransferase (COMT) and metabolized to Homovanillic acid by two further enzymatic reactions (44).

It has been well described by other authors that the DOPA metabolic pattern reflects the relative activities of the enzymes involved in the catecholamine synthesis pathway (45) and that the degree of enzyme activity is related to the maturation of cancer cells (46, 47). In mature tumors, norepinephrine, which is at a lower stage in the catecholamine metabolic pathway, is more expressed than dopamine and DOPA, which are earlier precursors of the pathway (**Supplementary Figure 3** and **Supplementary File 1**). A higher degree of maturation is generally considered to be related to a better prognosis.

Conversely, in poorly differentiated tumors, Aromatic L-amino acid decarboxylase (AAAD) and Dopamine beta-hydroxylase (DBH) enzymes may be relatively inactive causing an increase in dopamine and DOPA, being thus generally considered as biomarkers of unfavorable NB.

Recently, plasma 3-O-MD has also been included in a panel of L-DOPA metabolites analyzed by LC-MS/MS (43) in an attempt to assess whether metabolites produced in earlier steps of catecholamine metabolism might offer improved diagnostic accuracy over urinary HVA and VMA. Nevertheless, authors were only able to provide preliminary evidence that addition of 3-O-MD might be useful for diagnosis.

Since untargeted metabolomics is based on a semiquantitative approach and is unable to quantify metabolites with high accuracy (48), it was crucial for us to confirm our discoveries on a second cohort of patients by using a targeted analytical method. These evidences motivated our choice of conducting an additional validation of 3-O-MD. On the second cohort, we validated that 3-O-MD is an independent prognostic factor able to stratify clinical groups that, otherwise, can be hardly differentiated. From the differential expression analysis of metastatic patients at diagnosis and post induction chemotherapy, it was also evident that 3-O-MD was down-regulated in the second group, thereby suggesting its potential role as marker treatment response. In the same cohort, we assessed the ability of 3-O-MD to improve patients’ stratification, thus showing a clinical utility. The clinical utility of novel prognostic factors is often evaluated in selected groups of patients defined by combination of established NB risk factors. In the present study, we assessed the prognostic value of 3-O-MD in clinically relevant groups of NB patients whose stratification was rarely reported (3). This is the case of the subset of high-risk patients older than 18 months with metastatic (INRG stage M) disease. Patients belonging to this group suffer from the lowest survival probability and no risk factors are available for stratification in the INRG pre-treatment risk stratification schema yet (2), thereby defining them as one of the most challenging, from the clinical point of view. In this paper we demonstrated that 3-O-MD was additionally able to stratify this subset of patients, being thus an eligible biomarker to be included in the upcoming NB pre-treatment risk scheme.

Moreover, our approach was able to highlight other metabolites that do not belong to the L-DOPA catabolic pathway, but that might contribute to the diagnosis or prognosis of NB (**Supplementary Figure 4**).

In particular, 3-Hydroxyisobutyric acid and L-cystathionine were found to be more expressed in metastatic patients at diagnosis when compared to those after induction chemotherapy. 3-Hydroxyisobutyric acid is an intermediate in the metabolism of valine that has been found in elevated amounts in the urine of patients with various tumors (49, 50). Cystathionine is an intermediate in the synthesis of cysteine produced by the trans-sulfuration pathway which converts homocysteine into cystathionine (51).

A possible diagnostic role of L-cystathionine was postulated by Abeling et al. (45) raising the attention to a potential involvement of its pathway in NB.

Interestingly, in the comparison between metastatic patients at diagnosis and after induction chemotherapy, a number of metabolites of the methionine metabolism (52) were found in addition to L-cystathionine: methionine sulfoxide, adenosine and spermidine. Recent studies revealed the important role of methionine as a metabolic dependency of tumor-initiating cells and the association of the cancer cell growth with elevated methionine cycle activity (53–55).

By analyzing the differences between the same groups we also found, in accordance with Quintás et al. (13), two metabolites, spermine and spermidine, which belong to the family of polyamines. The presence of higher levels of polyamines could be associated with the mechanisms involved in tumor metastases in which polyamine synthetic pathway is a direct downstream target of several oncogenes including the MYC family (56).

A combined biomarker model might achieve a better sensitivity and specificity and should be implemented on a separate second cohort.

The point of strength of our approach over others, adopting tissue biopsies, is the lack of invasiveness of the sampling method that requires a limited volume of plasma. Moreover, the quantification of the validated biomarker, 3-O-MD, is reliable using LC-MS/MS, analytical technique that is being currently implemented in clinical reference laboratories for the routine biochemical assessment of NB patients at diagnosis.

## Limitation of This Study

The results of this study indicate that metabolomics is able to stratify NB patients and to provide clinically relevant biomarkers, however a number of limitations must be discussed. First of all, the sample size of the two cohorts was relatively small, further larger sample cohorts and multiple center study should be performed for more comprehensive validation.

Moreover, the survival analysis, that is the most clinically relevant conclusion of the study, could only be conducted in 75 patients with at least 3 years follow-up. In an attempt to deepen the analysis in clinically relevant subgroups the number of patients further decreased and, even if the statistically significance was guaranteed, the numbers were low.

In addition, even if a 3 years-follow-up is generally considered relevant in high-risk NB patients, a longer (5 years) follow-up time could be additionally considered in future analyses.

## Conclusion

In conclusion, in this study, LC-HRMS untargeted metabolomics was successfully used in an attempt to identify new prognostic biomarkers of metastatic NB. These results contribute new insights into metastatic NB and identify 3-O-MD as a novel biomarker of potential clinical relevance that can be reliably measured from a very small amounts of sample material. Further research in larger studies and external validation is warranted to determine the clinical applicability of this metabolic biomarker in the diagnosis of NB. Moreover, other metabolites that have been highlighted by our approach deserve further validation in subsequent studies on independent cohorts of patients.

## Data Availability Statement

The original contributions presented in the study are publicly available. This data can be found here: www.ebi.ac.uk/metabolights/MTBLS4294.

## Ethics Statement

The studies involving human participants were reviewed and approved by Ethics Review Board of IRCCS Istituto Giannina Gaslini (16 May 2004). Written informed consent to participate in this study was provided by the participants’ legal guardian/next of kin.

## Author Contributions

Conceptualization, SB, DC and GC. Data curation, SB and CL. Formal analysis, DC, LO and AP. Funding acquisition, LO. Investigation, SB. Methodology, PU and MC. Project administration, AP and GC. Resources, MM and AE. Supervision, PU, AG and GT. Validation, GC. Writing— original draft, DC, AP and GC. Writing—review and editing, SB, DC, MM, PU, MC, AP and GC. All authors have read and agreed to the published version of the manuscript. All authors contributed to the article and approved the submitted version.

## Funding

This study was funded by Fondazione Italiana per la Lotta al Neuroblastoma and the Italian Ministry of Health, RC2021. We gratefully thank all Italian citizens who allocated the 5 × 1000 share of their tax payment in support of health research.

## Conflict of Interest

The authors declare that the research was conducted in the absence of any commercial or financial relationships that could be construed as a potential conflict of interest.

## Publisher’s Note

All claims expressed in this article are solely those of the authors and do not necessarily represent those of their affiliated organizations, or those of the publisher, the editors and the reviewers. Any product that may be evaluated in this article, or claim that may be made by its manufacturer, is not guaranteed or endorsed by the publisher.
