# Supplementary material for: Untargeted LC-HRMS Based-Plasma Metabolomics Reveals 3-O-Methyldopa as a New Biomarker of Poor Prognosis in High-Risk Neuroblastoma
Source: Front Oncol. 2022 Jun 10;12:845936. doi: 10.3389/fonc.2022.845936 (PMC9231354; doi:10.3389/fonc.2022.845936)
Supplement: Supplementary file 1 [file DataSheet_1.docx]

Supplementary Material

# Supplementary Data

## Chemicals and consumables

Ammonium formate, acetonitrile (ACN), methanol and formic acid (FA), all LC-MS grade, were purchased from Sigma Aldrich Srl (Milan, Italy). Water was purified by reverse osmosis and filtered through a Milli-Q purification system (Millipore, Milford, MA, USA).

The mixture of internal standards used in metabolomics analysis contained: chenodeoxycholic acid (2,2,4,4-D4, 98%, DLM-6780-PK), uracil (1,3-15N2, 98%, NLM-637-PK), vitamin B3 (D4, 98%, DLM-6883-PK), creatine (METHYL-D3, 98%, DLM-1302-PK), L-asparagine (15N2, 98%, NLM-3286-0.25), L-cysteine (15N, 98%, NLM-2295-PK) and L-arginine (15N4, 98%, NLM-396-PK).

Internal standard kit (MSK-QC-KIT) contained the following molecules: L-Alanine (13C3, 99%), L-Leucine (13C6, 99%), L-Phenylalanine (13C6, 99%), L-Tryptophan (13C11, 99%), L-Tyrosine (13C6, 99%), Caffeine (13C3,99%), D-Glucose (13C6, 99%), Sodium benzoate (13C699%), Sodium citrate (13C3, 99%), Sodium octanoate (13 C8, 99%), Sodium propionate (13C3, 99%), Stearic acid, sodium salt (13 C18, 98%), Succinic acid disodium salt (13 C4, 99%), D-Sucrose (13 C6, 98%).

All molecules were purchased from Cambridge Isotope Laboratories, Inc (Tewksbury, MA, USA). Internal standard for target analysis was L-3-(4-Hydroxy-3-methoxy-d3-phenylalanine (D-6782) purchased from C/D/N Isotopes Inc. (Pointe-Claire, Quebec, Canada).

## Untargeted metabolomic analysis

The chromatographic columns and conditions used for the untargeted metabolomic analysis were the following:

Reversed phase gradient separation chromatography was carried out using a ACQUITY BEH C18 (2.1 mm X 100 mm, i.d. 1.7 µm, Waters, Milan, Italy) with mobile phase A consisting of 0.1% formic acid in water, and mobile phase B of 0.1 % formic acid in acetonitrile. The percentage of solvent B started at 1% for 0.1 min, reached 100% in 15 min and was kept for 5 min at flow rate of 250 μL/min, then the column was reconditioned at 1% B for 5 min for a total run time of 25 min. The column temperature was maintained at 40 °C.

HILIC phase gradient separation chromatography was carried out using a ACQUITY BEH Amide (2.1 mm X 150 mm, i.d. 1.7 µm, Waters, Milan, Italy) with mobile phase A consisting of 5 mM ammonium formate, pH 3, in water and mobile phase B of acetonitrile. The percentage of solvent B started at 10% for 0.1 min, reached 70% in 15 min and was kept for 5 min at flow rate of 200 μL/min, then the column was reconditioned at 1% B for 9 min for a total run time of 27 min. The column temperature was maintained at 25 °C.

## Targeted analysis of 3-O-methyldopa by LC-MS/MS

Gradient separation chromatography was carried out using a ACQUITY UPLC HSS PFP column (2.1 mm X 150 mm, i.d. 1.8 μm, Waters, Milan, Italy) with mobile phase A consisting of 0.1% formic acid in water, and mobile phase B of 0.1 % formic acid in acetonitrile. The percentage of solvent B started at 10% for 0.1 min, reached 98% in 1.4 min and was kept for 1 min at flow rate of 400 μL/min, then the column was reconditioned at 10% B for 2.5 min for a total run time of 5.3 min. The column temperature was maintained at 40 °C. Ionization was achieved using heated electrospray ionization with a spray voltage of 3500 V in positive mode. Nitrogen was used as the nebulizer and auxiliary gas, set at 60 and 10 arbitrary units, respectively. Vaporizer and ion transfer tube temperature were set at 350 °C and 400 °C. For collision-induced dissociation, high purity argon was used at a pressure of 1.5 mTorr. Analyte and IS were detected using selected reaction monitoring of the specific transitions: 3-O-methyldopa, 213.3 → 149; 3-O-methyldopa-d3, 215.1 → 152.

## Mass spectrometry methods validation and quality assurance

System suitability assessment for Untargeted method, such as retention time and peak areas have been monitored over time. The average coefficient of variation of the internal standards areas during 145 injections was 17% (Supplementary Table 5). Supplementary Figure 5 (panel A and B) shown the shift of areas and retention times of QCs over entire positive reverse phase analysis. For a representative isotopic molecule, the mass precision shift is reported (panel C), the average mass error never been exceeded 2.5 ppm. In Supplementary Figures 6-9 are shown principal component analysis (PCA) plot of all samples and QC in each experiment, in order to visually assess the difference in multivariate dispersion. The QC data points cluster tightly in comparison to the observed dispersion of biological samples, then these data can be deemed as of high quality in term of precision. Here, PCA analysis is intended for quality assurance during the untargeted approach not to demonstrate clustering of patients groups/stages.

The target method was validated following EMA guidelines for bioanalytical method validation in terms of selectivity, linearity, carry over, calibration curve, accuracy, precision and LLOQ. The results achieved showed excellent selectivity with no interfering peaks at the specified chromatographic conditions. Carry-over was absent. The LLOQ, intra- and inter- assay precision and accuracy were all inside the acceptable ranges are summarized in Supplementary Table 6. The linear regression fit for all the calibration curves (n=5) was achieved (with average r2 > 0.9988, Supplementary Figure 10). All back-calculated values did not differ from ± 15% of the theoretical value.

## Parameters of the data processing software

MS-DIAL parameters were set as follows: MS1 tolerance, 0.05Da; MS2 tolerance, 0.025 Da; retention time begin, 0 min; retention time end, 100 min; minimum peak height, 10000; mass slice width, 0.1 Da; smoothing level, 3 scans; minimum peak width, 5 scans; sigma window value, 0.5. We considered M−H, M–H2O−H, M+Na-2H, M+Cl, M+FA-H, 2M−H, 2M+FA-H, M−2H, 3M-H adduct in negative ionization mode and M+H, M+Na, M+ACN+H, M+H–H2O, M+H–2H2O, M+2Na-H, M+ACN+Na, M+2ACN+H, 2M+H, M+2H, 2M+ACN+Na in positive ionization mode. Execute retention time correction on IS and IS kit with a RT tolerance of 0.1 min and a mass tolerance of 0.015 Da were performed. MS-FINDER parameters were set as follows: The MS1 and MS2 tolerances were set to 5 and 15 ppm, respectively. Formula finder were exclusively processed with C, H, O, N, P and S atoms

# Supplementary Figures and Tables

## Supplementary Figures

## Supplementary Figure 1. Experimental design of untargeted metabolomic analysis and analytical workflow.

**
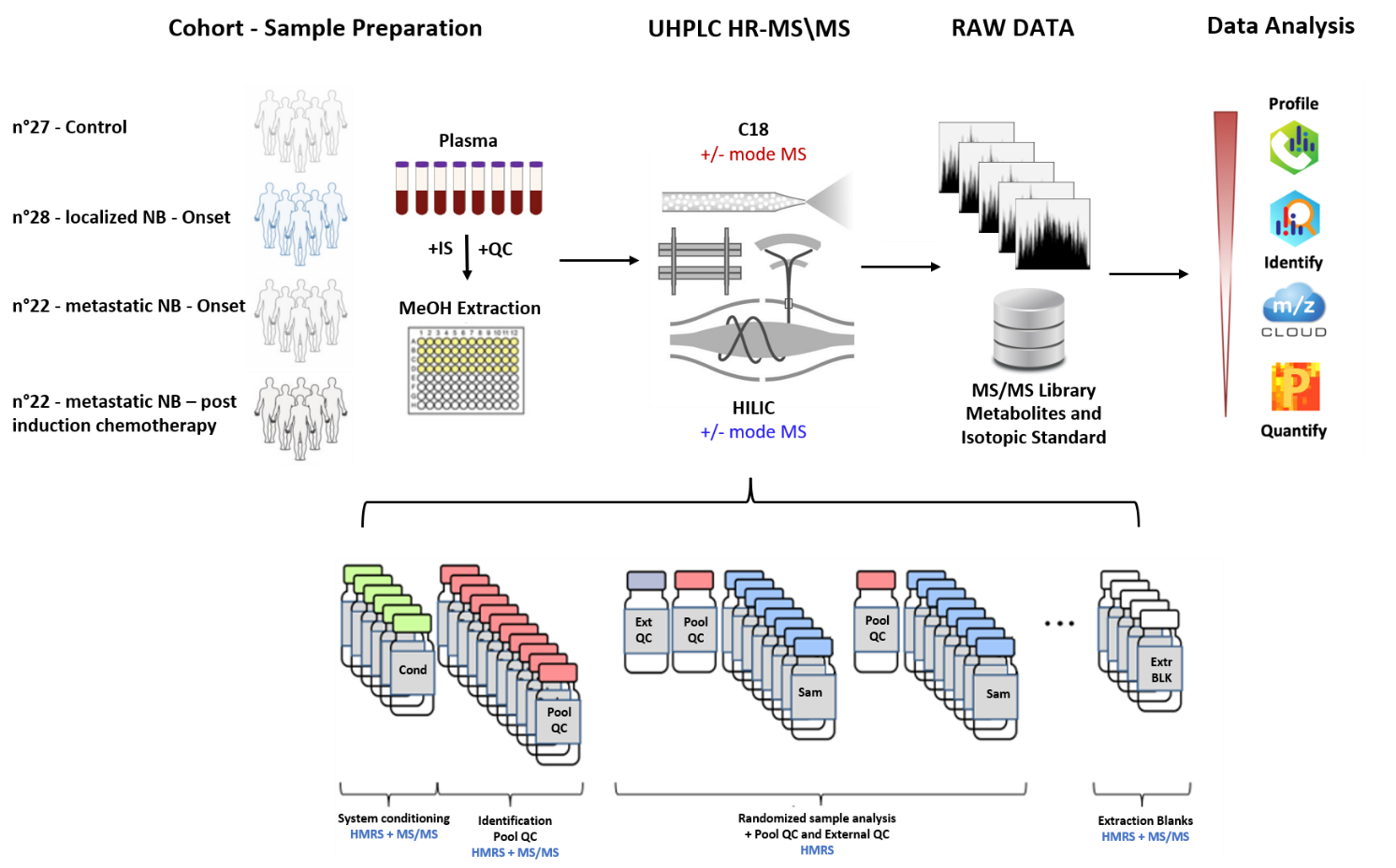
**

**Supplementary Figure 2.** ClueGO results: biological processes associated with statistically significant metabolites. For each metabolite found to be significant in the "Onset Metastatic vs Metastatic Post Chemotherapy" and "Onset Metastatic vs Onset Localized" comparisons, we collected, via the HMDB resource, interactions with enzymes and proteins. When multiple interactions were present, we chose to apply a binary method, one metabolite - one protein, to avoid over-representation bias. The selected proteins were then used to perform common enrichment analysis with FDR <= 0.01, GO Tree Interval 3-8, with a minimum gene number of 3. Color is associated with the GOgroup, and of each we graphically report the proteins, small spheres, and the associated pathway, large spheres.
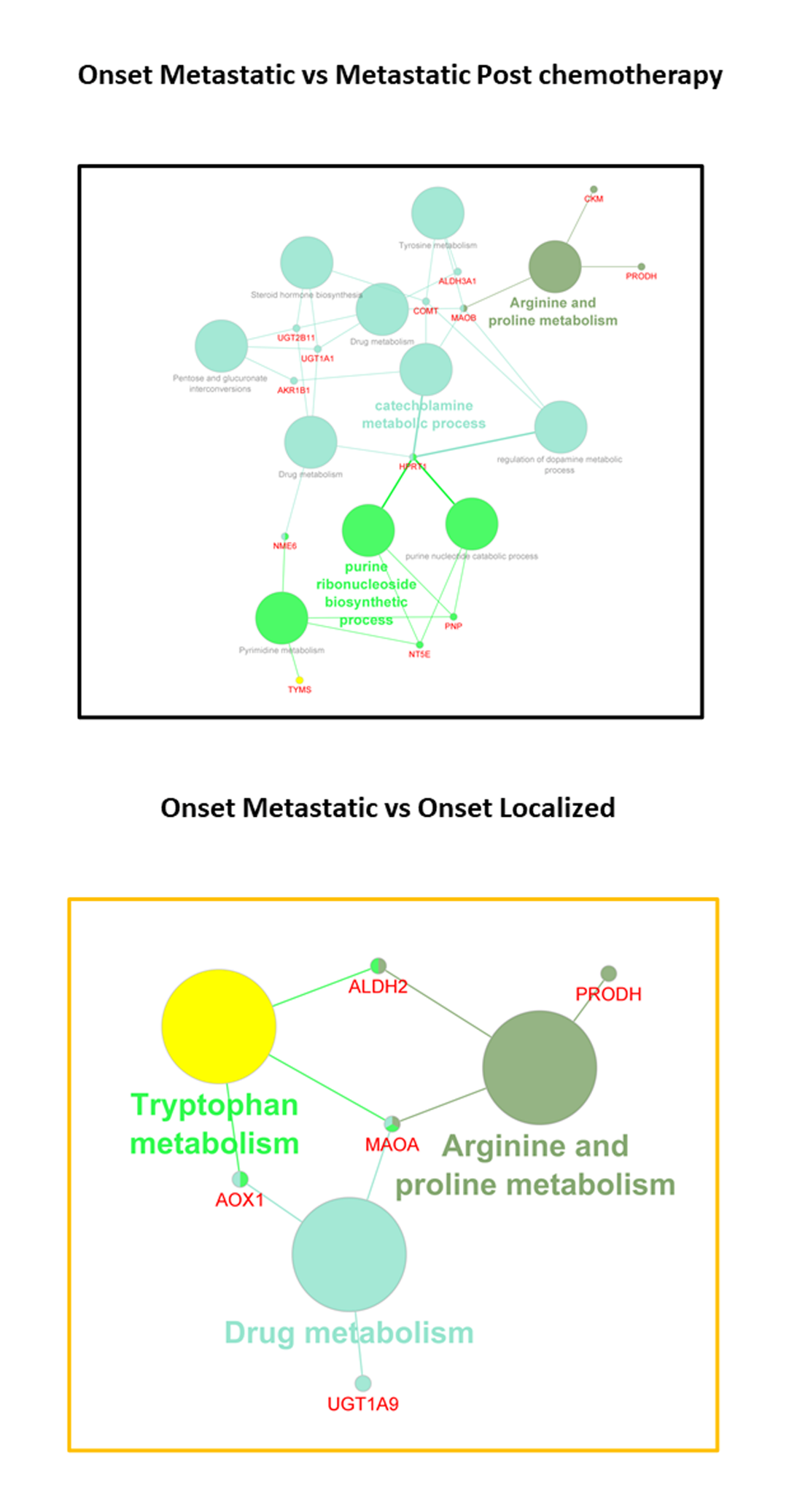


**Supplementary Figure 3.** Metabolism of L-DOPA. In Supplementary file are reported more information about pathway reactions, metabolites and enzymes.


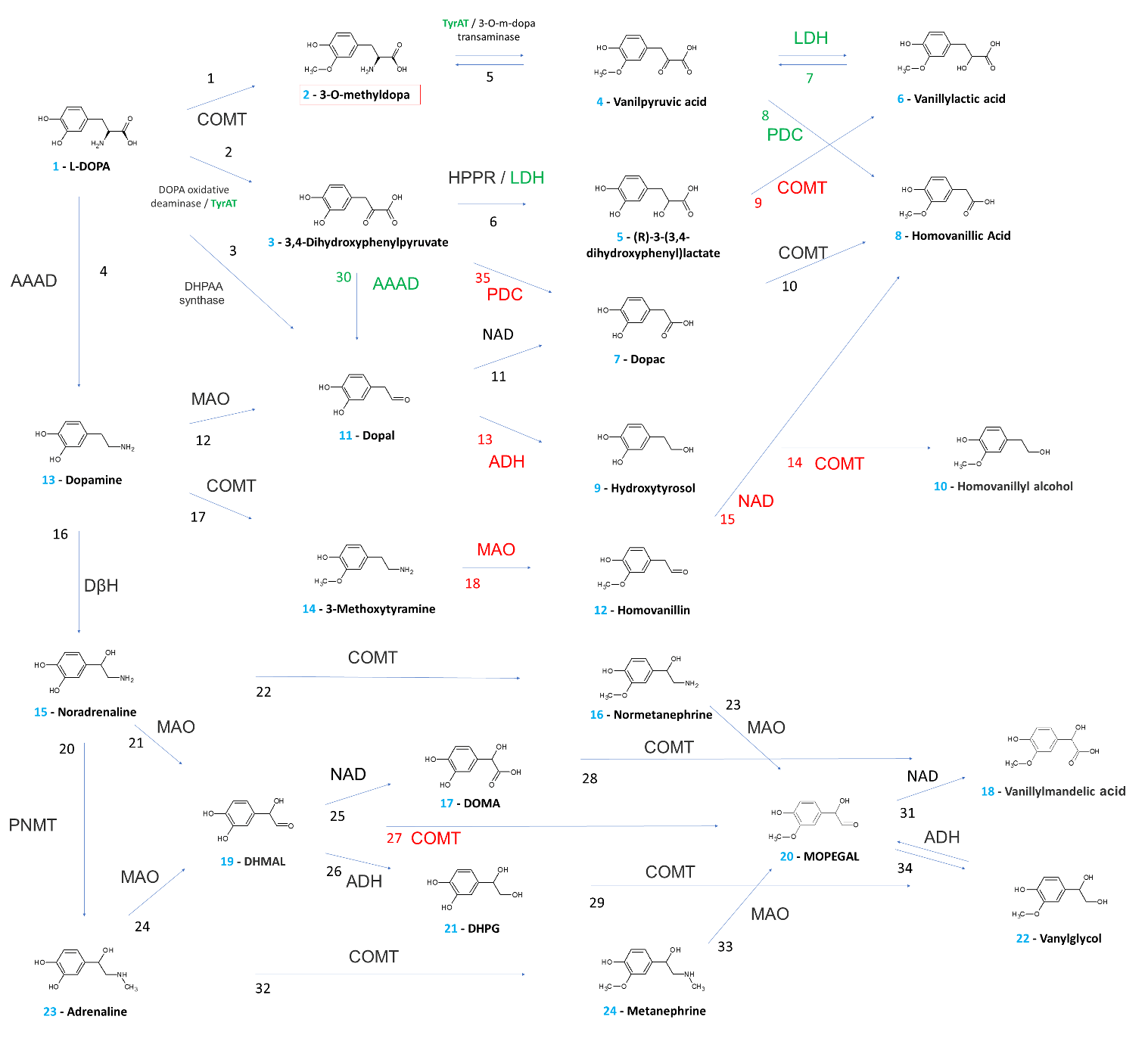


**Supplementary Figure 4.** MetaboAnalyst results, summary plot for over representation analysis of metabolic pathways: (A) Significant metabolites in healthy controls (B) Significant metabolites in all the patients at the onset (C) All significant metabolites in the comparison between metastatic and localized patients at the onset (D) All significant metabolites in the comparison between metastatic NB patients at the onset and after chemotherapy.

**
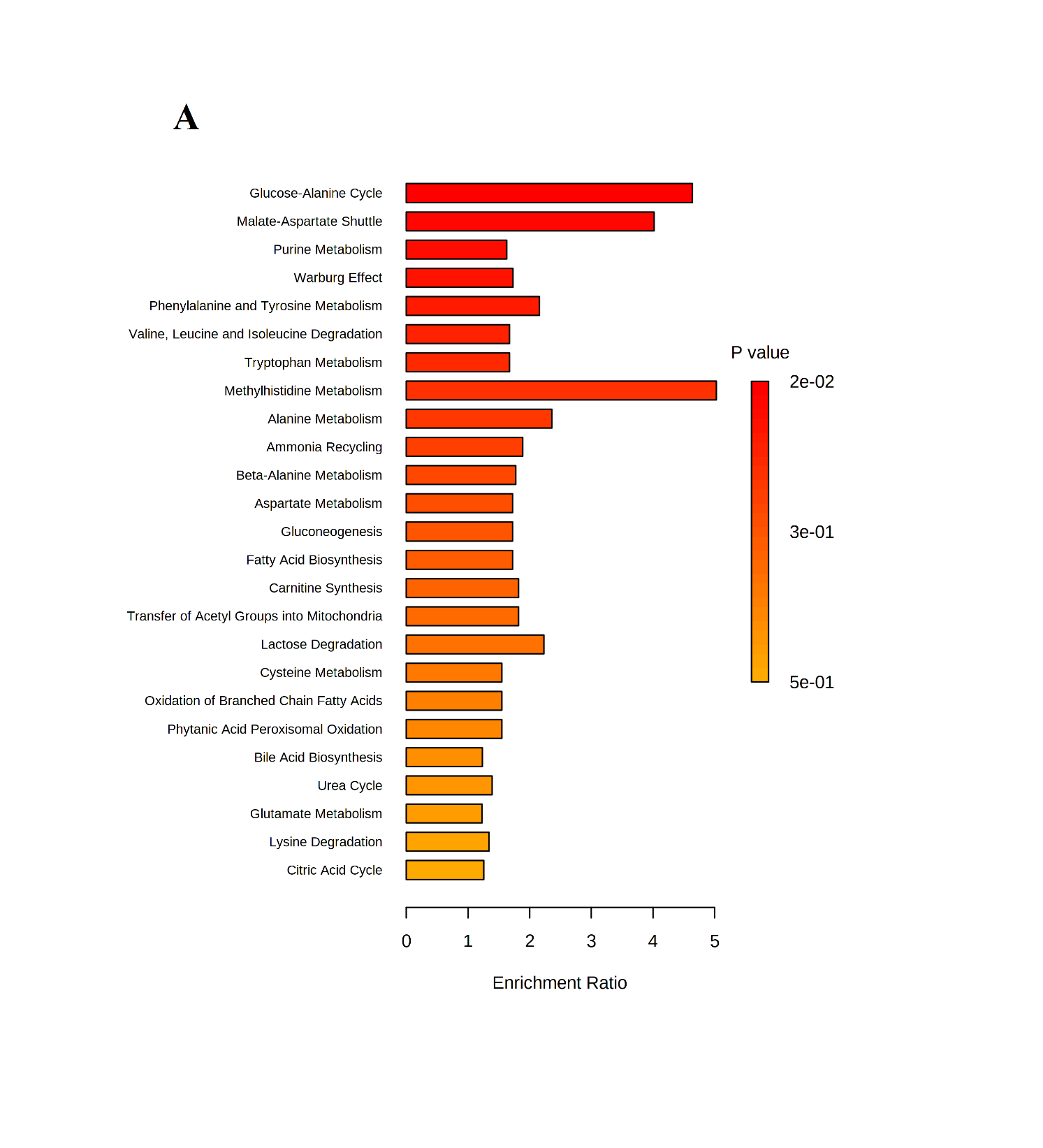
**

**
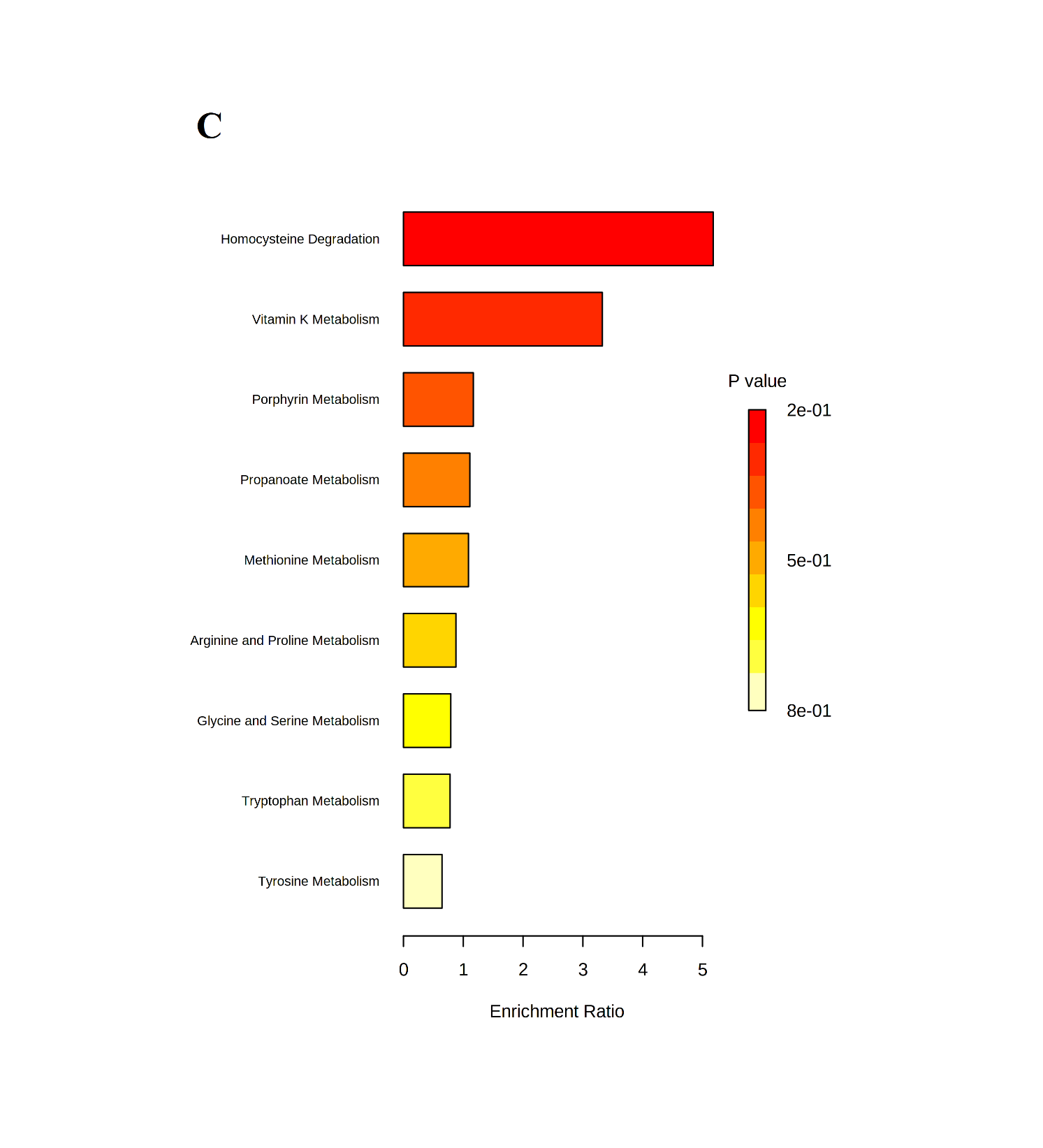
**

**
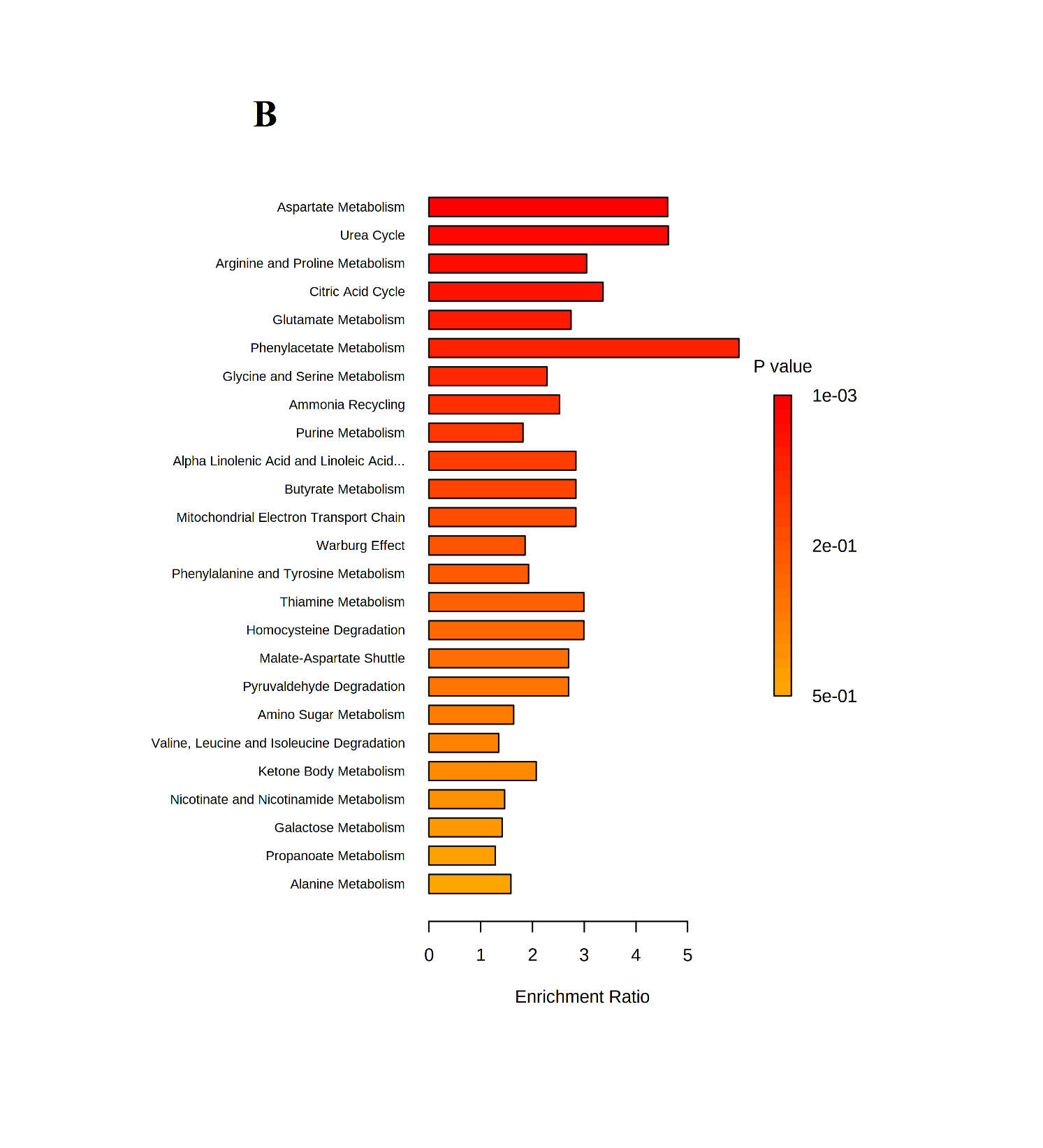
**

**
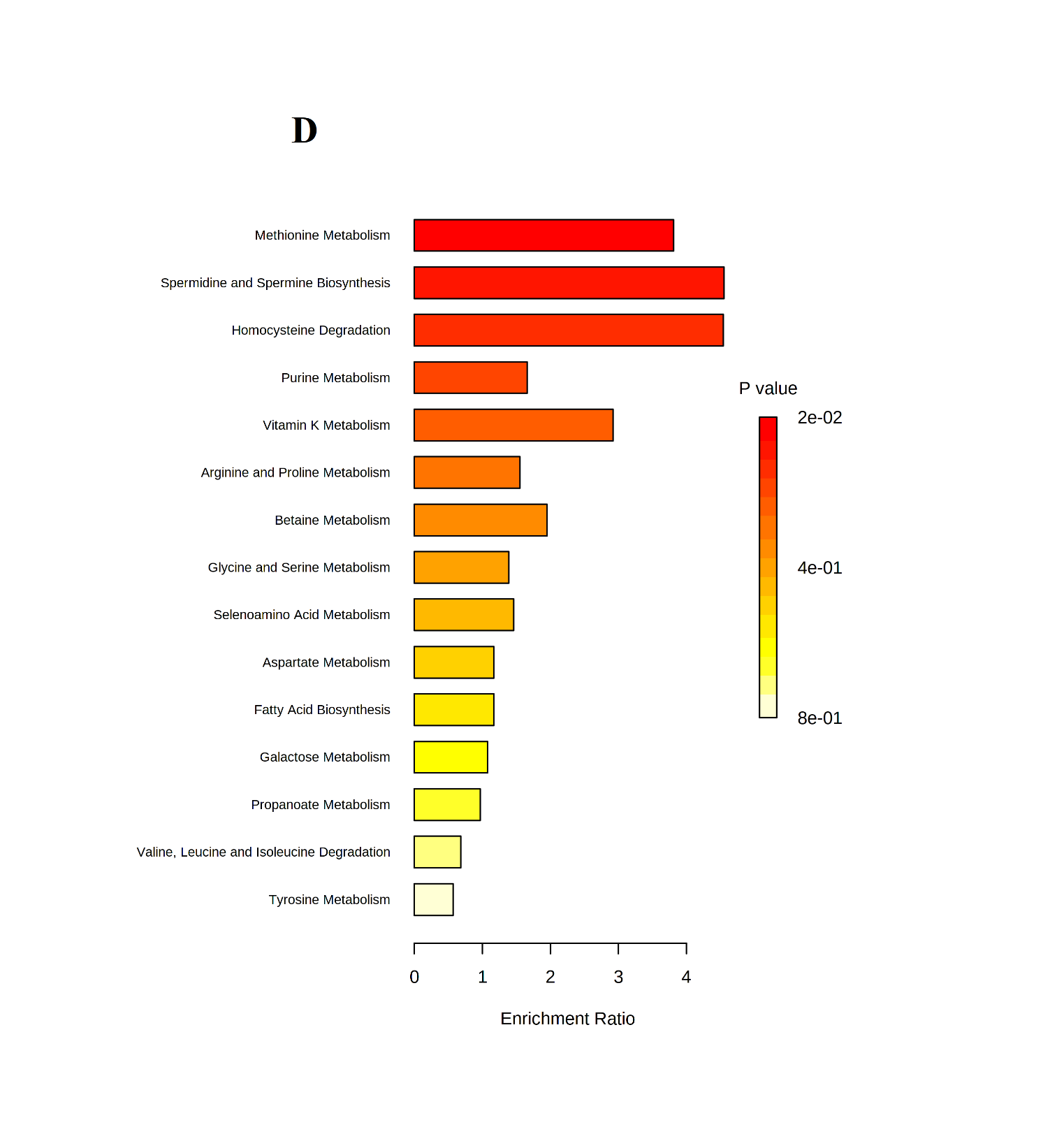
**

**Supplementary Figure 5.** Quality assurance of untargeted metabolomic analysis.

**
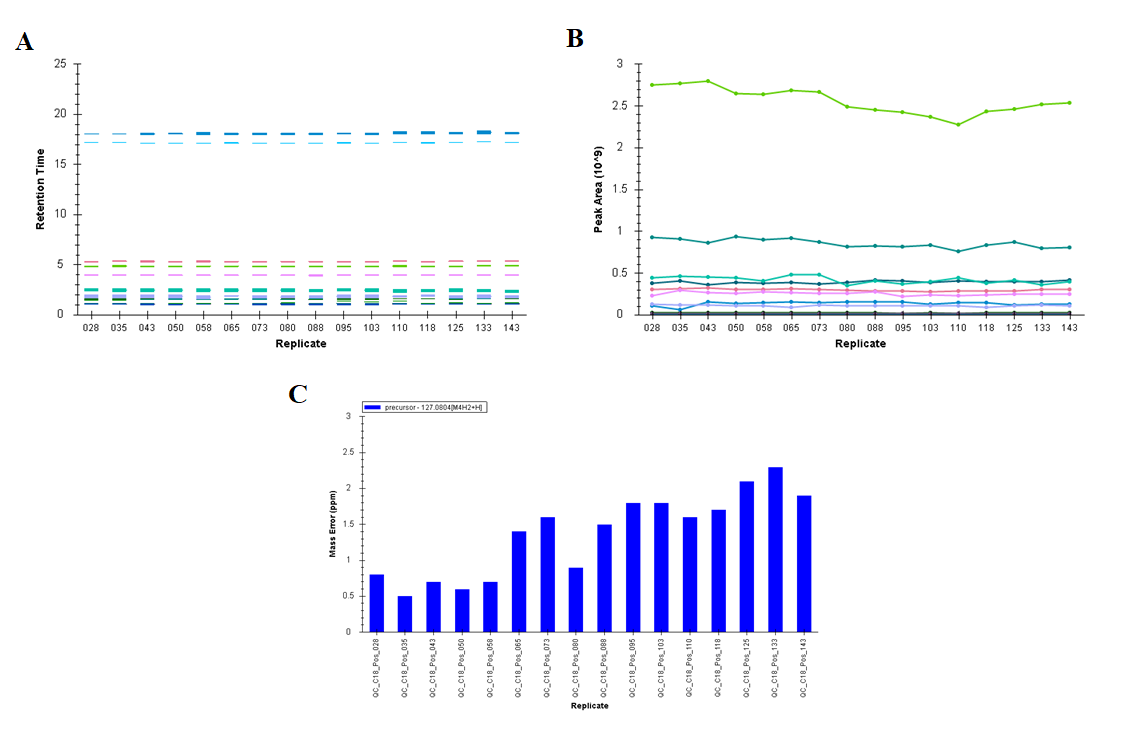
**

**Supplementary Figure 6. PCA plot of all samples and QC by C18 chromatography and positive ionization.**

**
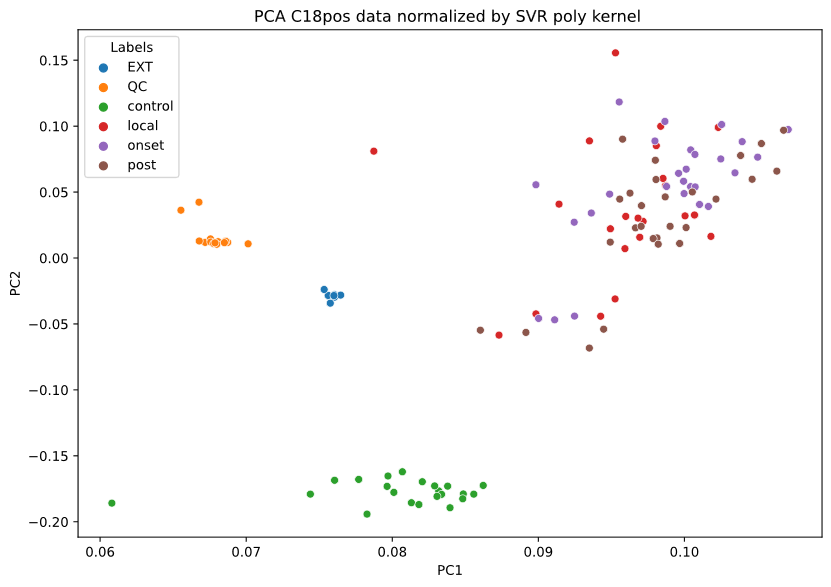
**

**Supplementary Figure 7. PCA plot of all samples and QC by C18 chromatography and negative ionization.**

**
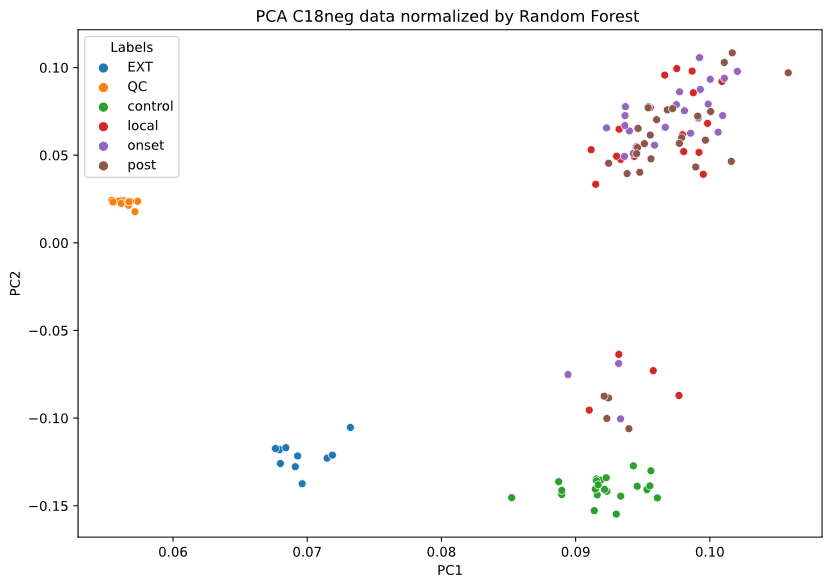
**

**Supplementary Figure 8.** **PCA plot of all samples and QC by HILIC chromatography and positive ionization.**

**
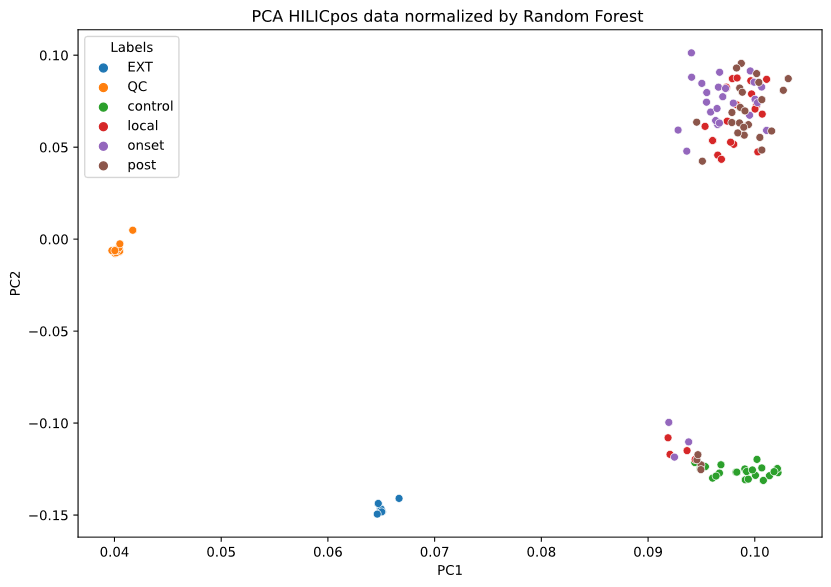
**

**Supplementary Figure 9. PCA plot of all samples and QC by HILIC chromatography and negative ionization.**

**
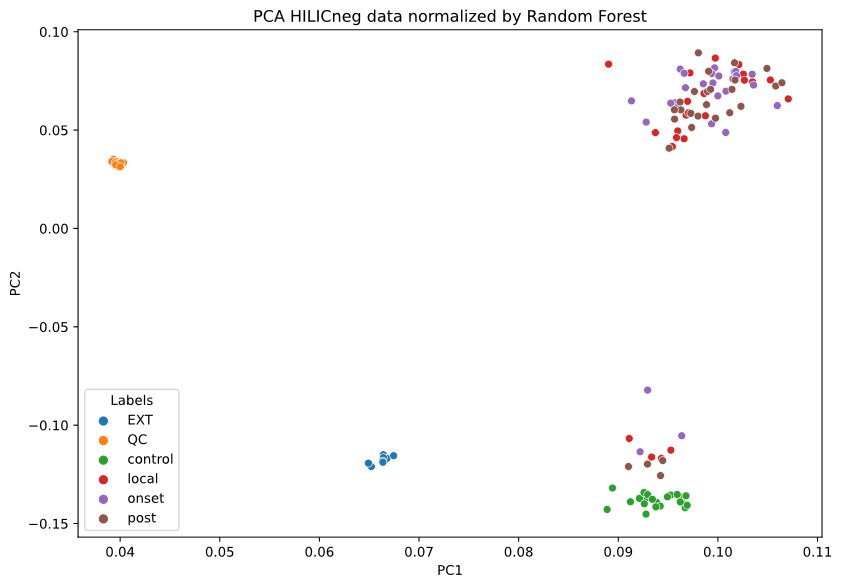
**

**Supplementary Figure 10.** Average calibration curves of targeted analysis of 3-O-methyldopa by LC-MS/MS.


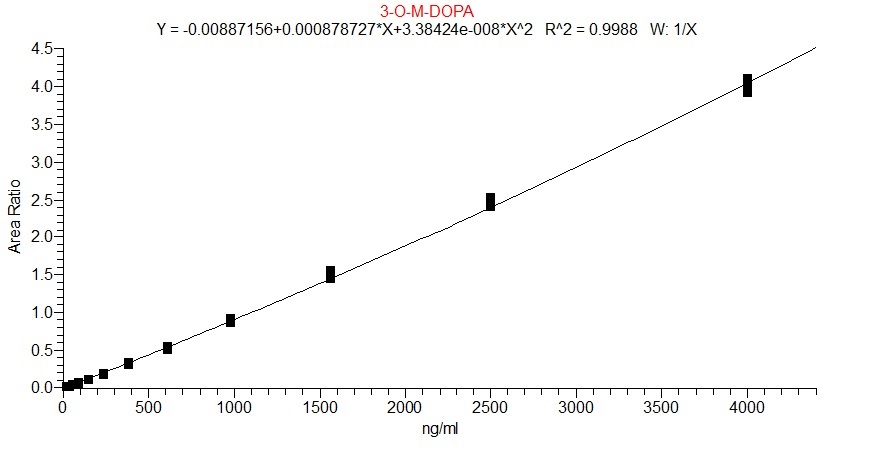


## Supplementary Tables

**Supplementary Table 1.** **Fischer's exact test results for evaluetion of confounding between CTR subjects and NB patients by Sex (Male vs Female) and Age group (<18 months vs >18 months).**

|  |  | Group | |  |
| --- | --- | --- | --- | --- |
|  |  | CTR | NB | P value |
|  |  |  |  |  |
| Sex | M | 12 | 21 | 0.4419 |
|  | F | 10 | 29 |  |
|  |  |  |  |  |
| Age | <18 | 5 | 21 | 0.1825 |
|  | >18 | 17 | 29 |  |
|  |  |  |  |  |

**Supplementary Table 2.** Significantly modulated metabolites by differential expression analysis between NB patients at the onset (positive FC) and controls (negative FC).

| **LogFC^a^** | **-Log**  **(adj.P-Val)**  **^b^** | **Common Name** | **Accession HMDB** | **LogFC^a^** | **-log**  **(adj.P-Val)**  **^b^** | **Common Name** | **Accession HMDB** |
| --- | --- | --- | --- | --- | --- | --- | --- |
| 6.91 | 15.45 | cis-Aconitic acid | HMDB0000072 | -2.17 | 8.61 | Myristic acid | HMDB0000806 |
| 6.62 | 35.81 | Bergaptol | HMDB0013679 | -2.19 | 3.38 | LysoPE(15:0/0:0) | HMDB0011502 |
| 6.54 | 6.49 | Pyroglutamic acid | HMDB0000267 | -2.22 | 4.37 | LysoPC(18:2(9Z,12Z)) | HMDB0010386 |
| 5.93 | 7.52 | 2-Hydroxy-3-methylbutyric acid | HMDB0000407 | -2.25 | 8.38 | 1,3-Dimethyluric acid | HMDB0001857 |
| 5.65 | 4.29 | p-Cresol sulfate | HMDB0011635 | -2.25 | 13.45 | 1-Methylxanthine | HMDB0010738 |
| 5.48 | 2.58 | Galactitol | HMDB0000107 | -2.35 | 4.15 | Phenol sulphate | HMDB0060015 |
| 5.42 | 2.32 | Inosinic acid | HMDB0000175 | -2.36 | 14.76 | Ketoleucine | HMDB0000695 |
| 5.14 | 6.44 | 15(S)-HETE | HMDB0003876 | -2.38 | 2.21 | Dehydroepiandrosterone sulfate | HMDB0001032 |
| 4.78 | 13.56 | Glucosamine 6-phosphate | HMDB0001254 | -2.39 | 18.81 | Malonic acid | HMDB0000691 |
| 4.73 | 8.96 | Fumaric acid | HMDB0000134 | -2.44 | 7.81 | Guanosine | HMDB0000133 |
| 4.43 | 2.63 | Adenosine monophosphate | HMDB0000045 | -2.44 | 11.79 | Xanthosine | HMDB0000299 |
| 4.32 | 3.45 | Sucrose | HMDB0000258 | -2.44 | 3.45 | Erythronic acid | HMDB0000613 |
| 4.19 | 20.14 | Citric acid | HMDB0000094 | -2.49 | 4.64 | Uric acid | HMDB0000289 |
| 3.77 | 11.54 | Docosahexaenoic acid | HMDB0002183 | -2.49 | 9.12 | Kynurenic acid | HMDB0000715 |
| 3.75 | 6.15 | 2-Aminoheptanoate | HMDB0094649 | -2.53 | 13.58 | L-Lactic acid | HMDB0000190 |
| 3.68 | 2.99 | Piperidine | HMDB0034301 | -2.54 | 33.48 | Oxoglutaric acid | HMDB0000208 |
| 3.58 | 10.60 | D-2-Hydroxyglutaric acid | HMDB0000606 | -2.58 | 4.79 | Indoxyl sulfate | HMDB0000682 |
| 3.28 | 12.22 | 3-Dehydrocarnitine | HMDB0012154 | -2.58 | 5.10 | Glycocholic acid | HMDB0000138 |
| 3.25 | 6.15 | Pyruvaldehyde | HMDB0001167 | -2.61 | 15.09 | gamma-Aminobutyric acid | HMDB0000112 |
| 3.18 | 5.53 | L-Palmitoylcarnitine | HMDB0000222 | -2.61 | 20.79 | Hypoxanthine | HMDB0000157 |
| 3.02 | 9.57 | Thymine | HMDB0000262 | -2.63 | 5.00 | Leucyl-Isoleucine | HMDB0028932 |
| 2.93 | 23.52 | 2,5-Dioxopentanoate | HMDB0060365 | -2.67 | 22.86 | L-Glutamic acid | HMDB0000148 |
| 2.84 | 7.98 | Linoleic acid | HMDB0000673 | -2.72 | 3.90 | Taurocholic acid | HMDB0000036 |
| 2.83 | 3.90 | L-Cystathionine | HMDB0000099 | -2.74 | 9.12 | N-Acetylornithine | HMDB0003357 |
| 2.68 | 12.96 | L-Threonine | HMDB0000167 | -2.79 | 2.61 | Trimethylamine N-oxide | HMDB0000925 |
| 2.67 | 8.42 | Galactonic acid | HMDB0000565 | -2.83 | 17.69 | L-Tryptophan | HMDB0000929 |
| 2.55 | 9.57 | N2-gamma-Glutamylglutamine | HMDB0011738 | -2.85 | 8.35 | Mannose 6-phosphate | HMDB0001078 |
| 2.47 | 6.63 | Glutaric acid | HMDB0000661 | -2.90 | 14.26 | Glyceric acid | HMDB0000139 |
| 2.46 | 5.19 | L-Proline | HMDB0000162 | -2.93 | 7.81 | Pyridoxal | HMDB0001545 |
| 2.43 | 6.11 | L-Glutamine | HMDB0000641 | -2.96 | 14.05 | L-Histidine | HMDB0000177 |
| 2.42 | 7.57 | L-Gulonolactone | HMDB0003466 | -3.04 | 6.66 | Chenodeoxycholic acid glycine conjugate | HMDB0000637 |
| 2.17 | 8.79 | Pipecolic acid | HMDB0000070 | -3.07 | 6.42 | Allantoin | HMDB0000462 |
| 2.06 | 12.22 | Succinic acid | HMDB0000254 | -3.10 | 5.53 | Tauroursodeoxycholic acid | HMDB0000874 |
| 0.87 | 18.44 | Itaconic acid | HMDB0002092 | -3.16 | 6.11 | Taurodeoxycholic acid | HMDB0000896 |
| 0.80 | 25.07 | L-Aspartic acid | HMDB0000191 | -3.21 | 4.79 | Inosine | HMDB0000195 |
| 0.55 | 18.90 | L-Valine | HMDB0000883 | -3.26 | 11.29 | L-Phenylalanine | HMDB0000159 |
| 0.54 | 7.28 | L-Arginine | HMDB0000517 | -3.31 | 3.35 | Ascorbic acid | HMDB0000044 |
| 0.44 | 17.57 | L-Isoleucine | HMDB0000172 | -3.39 | 8.79 | Uridine | HMDB0000296 |
| -2.01 | 2.45 | Isohyodeoxycholic acid | HMDB0000664 | -3.55 | 9.85 | (S)-3-Hydroxyisobutyric acid | HMDB0000023 |
| -2.03 | 4.61 | 3-Carboxy-4-methyl-5-propyl-2-furanpropionic acid | HMDB0061112 | -3.58 | 6.49 | 1,2-Diacylglycerol-Bile-PC-pool | HMDB0062267 |
| -2.10 | 17.34 | D-Glucose | HMDB0000122 | -3.68 | 5.93 | LysoPE(16:0/0:0) | HMDB0011503 |
| -2.11 | 7.52 | L-Kynurenine | HMDB0000684 | -4.21 | 5.88 | D-Leucic acid | HMDB0000624 |
| -2.12 | 10.25 | L-Malic acid | HMDB0000156 | -5.68 | 13.94 | 2-Acetolactate | HMDB0006833 |
| -2.13 | 16.77 | Methoxsalen | HMDB0014693 | -7.31 | 49.04 | Butyric acid | HMDB0000039 |
| -2.17 | 3.80 | Mercaptopurine | HMDB0015167 | -9.72 | 68.77 | Portulacaxanthin II | HMDB0012281 |
| ^a^ LogFC: fold change in logarithmic scale; ^b^-Log (adj.P-Val): adjusted p-value in negative logarithmic scale | | | | | | | |

**Supplementary Table 3.** Significantly modulated metabolites by differential expression analysis between localized (INRG stage L1 and L2, negative FC) and metastatic (INRG stage M, positive FC)

| **LogFC^a^** | **-log(adj.P-Val)^b^** | **Common Name** | **Accession HMDB** |
| --- | --- | --- | --- |
| -2.65 | 20.70 | (Cyclohexylmethyl)pyrazine | HMDB0036175 |
| -2.00 | 1.51 | Prolylhydroxyproline | HMDB0006695 |
| -3.21 | 5.99 | 5-(10,13-Nonadecadienyl)-1,3-benzenediol | HMDB0039867 |
| -2.00 | 3.50 | LysoPC(18:3(6Z,9Z,12Z)) | HMDB0010387 |
| -2.27 | 1.38 | Bilirubin | HMDB0000054 |
| -2.04 | 2.27 | PA(20:1(11Z)/15:0) | HMDB0115093 |
| -2.48 | 2.98 | 13-L-Hydroperoxylinoleic acid | HMDB0003871 |
| -2.11 | 1.33 | Tetrahydroaldosterone-3-glucuronide | HMDB0010357 |
| -3.67 | 1.34 | 4-Hydroxy-L-glutamic acid | HMDB0002273 |
| -2.89 | 2.49 | Indoleacetaldehyde | HMDB0001190 |
| -2.48 | 2.53 | Cotinine | HMDB0001046 |
| -2.56 | 1.40 | 2,6-Di-tert-butylbenzoquinone | HMDB0013817 |
| -3.36 | 1.34 | Chorismate | HMDB0012199 |
| -2.78 | 3.66 | Metanephrine | HMDB0004063 |
| -2.27 | 2.57 | Nicotine glucuronide | HMDB0001272 |
| -2.57 | 1.34 | LysoPE(15:0/0:0) | HMDB0011502 |
| 3.32 | 1.48 | 3alpha,4,7,7alpha-Tetrahydro-1H-isoindole-1,3(2H)-dione | HMDB0033903 |
| 2.29 | 2.13 | 3-Methoxytyrosine | HMDB0001434 |
| 2.03 | 1.38 | Curcumin | HMDB0002269 |
| 4.02 | 2.03 | 2-Methoxyacetaminophen sulfate | HMDB0062550 |
| 2.91 | 3.16 | Homovanillic acid sulfate | HMDB0011719 |
| 2.54 | 3.29 | Propionic acid | HMDB0000237 |
| 4.35 | 16.00 | L-Cystathionine | HMDB0000099 |
| 3.39 | 2.95 | 2-Thiophenecarboxaldehyde | HMDB0029717 |
| 3.59 | 1.46 | Dehydrocyanaropicrin | HMDB0035029 |
| 2.16 | 3.16 | 2-Hydroxy-3-methylbutyric acid | HMDB0000407 |
| ^a^ LogFC: fold change in logarithmic scale; ^b^-Log (adj.P-Val): adjusted p-value in negative logarithmic scale | | | |

**Supplementary Table 4.** Significantly modulated metabolites by differential expression analysis between metastatic patients at diagnosis (negative FC) and after induction chemotherapy (positive FC).

| **LogFC^a^** | **P Value** | **Common Name** | **Accession HMDB** |
| --- | --- | --- | --- |
| -2.34 | 8.76E-06 | Spermidine | HMDB0001257 |
| -5.18 | 2.44E-07 | 3alpha,4,7,7alpha-Tetrahydro-1H-isoindole-1,3(2H)-dione | HMDB0033903 |
| -2.44 | 1.36E-04 | Spermine | HMDB0001256 |
| -2.43 | 6.45E-06 | 3-Methoxytyrosine | HMDB0001434 |
| -2.42 | 1.73E-03 | 3-beta-Hydroxy-4-beta-methyl-5-alpha-cholest-7-ene-4-alpha-carbaldehyde | HMDB0059643 |
| -2.79 | 2.39E-06 | (R)-3-Hydroxyisobutyric acid | HMDB0000023 |
| -2.82 | 6.54E-06 | Methionine sulfoxide | HMDB0002005 |
| -2.26 | 9.11E-08 | 2-Hydroxyxanthone | HMDB0032997 |
| -3.49 | 9.07E-08 | Homovanillic acid sulfate | HMDB0011719 |
| -2.49 | 2.64E-04 | Ucriol | HMDB0036705 |
| -4.15 | 1.05E-04 | dIDP | HMDB0003536 |
| -2.09 | 4.56E-04 | Gabapentin | HMDB0005015 |
| -2.71 | 1.69E-07 | Propionic acid | HMDB0000237 |
| -2.13 | 8.47E-06 | D-Proline | HMDB0000162 |
| -2.40 | 2.74E-03 | Guanosine | HMDB0000133 |
| -2.11 | 1.19E-05 | Hypoxanthine | HMDB0000157 |
| -2.65 | 9.53E-05 | Vanillylmandelic acid | HMDB0133489 |
| -2.33 | 7.93E-04 | Syringic acid | HMDB0002085 |
| -2.91 | 7.69E-05 | Galactitol | HMDB0000107 |
| -5.11 | 1.94E-22 | L-Cystathionine | HMDB0000099 |
| -4.30 | 1.03E-04 | Furazolidone | HMDB0014752 |
| -3.25 | 4.08E-04 | Adenosine | HMDB0000050 |
| -3.59 | 2.08E-04 | Malonic acid | HMDB0000691 |
| -2.13 | 8.96E-04 | Creatine | HMDB0000064 |
| 5.24 | 1.52E-08 | 2-Phenylethanol glucuronide | HMDB0010350 |
| 2.06 | 7.68E-04 | 2-Pyrocatechuic acid | HMDB0000397 |
| 3.32 | 3.47E-04 | 4-Vinylphenol sulfate | HMDB0062775 |
| 2.30 | 1.53E-04 | Pyrogallol-1-O-sulphate | HMDB0060016 |
| 5.68 | 3.42E-08 | Trimethoprim | HMDB0014583 |
| 3.47 | 8.77E-06 | 5-Sulfoxymethylfurfural | HMDB0059752 |
| ^a^ LogFC: fold change in logarithmic scale | | | |

**Supplementary Table 5.** Coefficient of variation of isotope-labeled internal standards along non-targeted metabolomics analysis.
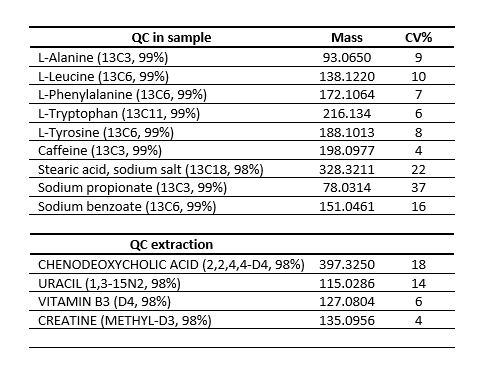


**Supplementary Table 6.** Intra and inter-assay coefficient of variation and accuracy of quality control samples for the targeted method validation.

**
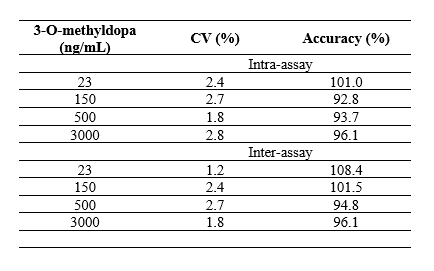
**
